# Durum Wheat Grain Yield and Quality under Low and High Nitrogen Conditions: Insights into Natural Variation in Low- and High-Yielding Genotypes

**DOI:** 10.3390/plants9121636

**Published:** 2020-11-24

**Authors:** Sinda Ben Mariem, Jon González-Torralba, Concha Collar, Iker Aranjuelo, Fermín Morales

**Affiliations:** 1 Instituto de Agrobiotecnología (IdAB), CSIC-Gobierno de Navarra, Avda. de Pamplona 123, 31192 Mutilva, Spain; sinda.ben@csic.es (S.B.M.); jon.gonzalez@unavarra.es (J.G.-T.); iker.aranjuelo@csic.es (I.A.); 2 Cereals and Cereal-Based Products, Food Science Department, Instituto de Agroquímica y Tecnología de Alimentos (CSIC), Avda. Catedrático Agustín Escardino, 7, 46980 Paterna, Spain; ccollar@iata.csic.es

**Keywords:** durum wheat yield, grain quality, nitrogen fertilization, *Triticum turgidum* L. var. *durum*

## Abstract

The availability and management of N are major determinants of crop productivity, but N excessive use has an associated agro-ecosystems environmental impact. The aim of this work was to investigate the influence of N fertilization on yield and grain quality of 6 durum wheat genotypes, selected from 20 genotypes as high- and low-yielding genotypes. Two N levels were applied from anthesis to maturity: high (½ Hoagland nutrient solution) and low (modified ½ Hoagland with one-third of N). Together with the agronomic characterization, grain quality analyses were assessed to characterize carbohydrates concentration, mineral composition, glutenin and gliadin concentrations, polyphenol profile, and anti-radical activity. Nitrogen supply improved wheat grain yield with no effect on thousand-grain weight. Grain soluble sugars and gluten fractions were increased, but starch concentration was reduced, under high N. Mineral composition and polyphenol concentrations were also improved by N application. High-yielding genotypes had higher grain carbohydrates concentrations, while higher concentrations in grain minerals, gluten fractions, and polyphenols were recorded in the low-yielding ones. Decreasing the amount of N to one-third ensured a better N use efficiency but reduced durum wheat agronomic and quality traits.

## 1. Introduction

Cereals are the most abundant field crops globally and considered as staple foods for humanity. Wheat is one of the most important cereal crops with an outstanding role in worldwide population nutrition [1]. Currently, *Triticum* grains contribute largely to human diet by providing carbohydrates, protein, dietary fiber, minerals, vitamins, and also phenolic acids that complement a balanced diet by their anti-oxidative potential [2]. Durum wheat (*Triticum turgidum* L. var. *durum*) represents 8% of the whole area cultivated with wheat and about 5% of world wheat production [3]. In spite of being relatively less important than bread wheat (*Triticum aestivum* L.), durum wheat is cultivated in many areas of the Mediterranean basin as a main cereal crop widely used for making pasta [4]. In fact, considering its cooking quality, durum wheat flour is technologically a preferred raw material for this purpose [5].

Wheat grain yield and quality is determined by genotype, environment and the interaction between them [6]. Among the environmental factors that affect strongly crop productivity and nutritional quality of cereal crops, fertilizers management is an important factor to obtain high yield and high quality harvests [7]. Nitrogen is one of the major nutritional elements required for adequate plant growth; hence, fertilization with N increases grain yields and improves end use quality [8,9,10]. As a consequence, N fertilizers application has been dramatically increased [11]. However, it has been estimated that only 30 to 40% of the applied N is absorbed by the crop and harvested in the grain [12]; thus, the excessive application leads to a huge loss of N contributing thereby to environment pollution. Therefore, an optimized N fertilization, i.e., a rational use of N fertilizers, is an important task for agronomists to improve crop N uptake, increase nitrogen use efficiency (NUE) and yields [13,14].

Increasing wheat yield has often been associated with grain quality losses. Research has been focused to investigate the relationship between N and grain protein. In this regard, different observations have been reported regarding grain protein content and composition depending on the rate of N and the timing of application. Nitrogen supply increased N accumulation in bread wheat grains, which enhanced protein content [15], resulting in an increase in both gliadins and glutenins [16]. Similar results were found by Abedi et al. [17], but an over-dose N application decreased wheat seed protein content. Late application of N resulted in gluten enhancement [17]. Makowska et al. [18] found a positive correlation between N dose and protein content, as well as glutenin in durum wheat grains. They claimed also that fertilization level influenced gluten proteins properties.

Conversely, less attention has been devoted in the literature to assess the effect of N fertilization on wheat grain carbohydrates. Regulation of metabolic processes by sugars depends on N supply, suggesting that N and sugar signaling pathways interact [19]. In fact, N metabolism requires C sources and energy from C metabolism, whereas C metabolism requires N metabolism to provide N-containing compounds, such as photosynthetic pigments and enzymes. Thus, the application of N fertilizer has indeed a significant effect on crop non-structural carbohydrate content [20]. Furthermore, C assimilation, allocation, and partitioning are strongly influenced by N supply affecting as a consequence carbohydrate distribution within the plant [21]. In the same way, Pan [22] found that, under low N conditions, the concentration and apparent transferred mass of non-structural carbohydrates were higher than those under high N conditions.

Mineral composition and content in wheat grain are also impacted by N fertilization. Numerous studies have shown that N application can promote the accumulation of some macro- and micro-elements in wheat grain [23,24,25], whereas they declined in other studies [26], showing that grain nutrient composition is controlled in part by genotype and in part by environmental factors. Adding to that, Dolijanovic et al. [7] found that an appropriate rate of N fertilizer has better impact on the concentration of macro- and micro-nutrients in wheat grain than using over-doses.

Phenolic compounds are secondary plant metabolites with strong antioxidant activity [27]. Ma et al. [28] indicated that N fertilization and irrigation have positive effects on wheat grain phenolic content and antioxidant activity. Other studies showed that wheat grain antioxidant properties are influenced by genotype, environment and genotype-environment interaction [29,30].

A crucial first step in any genetic mapping and breeding approach is to identify the existing variability in available germplasm. Genetic differences in NUE have been reported in the past in target crops, such as wheat. However, the molecular knowledge governing genetic variation among varieties in changing environmental conditions is still incomplete. In this work, a collection of 20 durum wheat varieties were provided by the International Maize and Wheat Improvement Center (CIMMYT, Mexico), which were selected in field conditions from a set of 120 genotypes as the ones having higher grain yield, protein, and starch concentrations under stressful growth conditions.

Taking into account the above-mentioned background, the aim of this work was to elucidate the response of durum wheat yield and grain quality to N fertilization. Towards this aim, we investigated the effects of two N levels in the fertilizer composition on grain yield and quality in different genotypes of durum wheat.

## 2. Results

### 2.1. Wheat Yield Components: Grain Yield and Thousand-Grain Weight

Significant differences were detected regarding grain yield and thousand-grain weight (TGW) among the 20 wheat genotypes fertilized by the two N levels (Table 1). This comparison allowed selecting 6 genotypes for grain quality analysis. Genotypes 18, 6 and 10 were selected as high-yielding genotypes since they recorded the highest values of grain yield and TGW under the two N levels, while genotypes 3, 9, and 16 showed the lowest values. It was surprising to see results of genotypes 20 and especially 17, which had higher grain yield under low than under high N. We are not sure if it could be related with pot placement in the greenhouse because pots were placed according to a randomized complete block design. Causes for that behavior in genotypes 20 and 17 were not further explored. Overall, high N fertilization treatment had significant positive effect on mean grain yield per plant, while no effect was detected on TGW (Table 1). Therefore, these data suggest that the increase of wheat grain yield observed under the high N treatment can be due to the increase in grain number produced per plant. The interaction genotype-treatment was not significant (Table 1).

### 2.2. Wheat Grain Nitrogen and Carbon Concentrations

Results presented in Table 2 show that the high N treatment significantly increased grain N and C concentrations by 29.24% and 2.43%, respectively. These changes led to a significant decrease in the carbon/nitrogen (C/N) ratio (Table 2). Regarding to genotype effect, the comparison among genotypes revealed that, generally, averages of grain N and C concentration were higher in the low-yielding genotypes (3, 9, and 16) (Table 2). Adding to that, NUE was significantly increased for plants grown under low N treatment and, as predicted, high-yielding genotypes (18, 6, and 10) had on average the highest values (Table 2).

### 2.3. Wheat Grain Carbohydrates Composition

**Figure 1:**
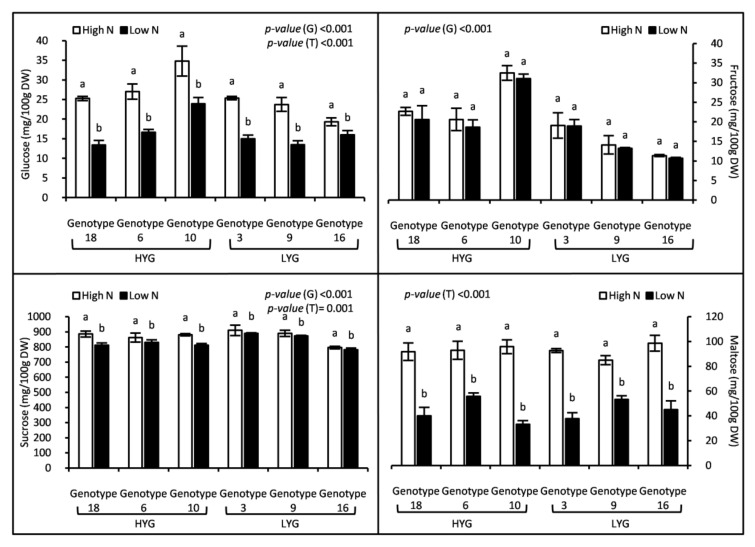
Grain soluble sugars concentrations of 6 wheat genotypes fertilized by two nitrogen levels (High and Low). Data correspond to the means ± standard error (n = 4). For each genotype, letters indicate significant differences between the two treatments at *p* < 0.5. *p*-values corresponds to two-way ANOVA analysis. HYG: High-yielding genotypes, LYG: Low-yielding genotypes, G: Genotype, T: Treatment.

**Figure 2:**
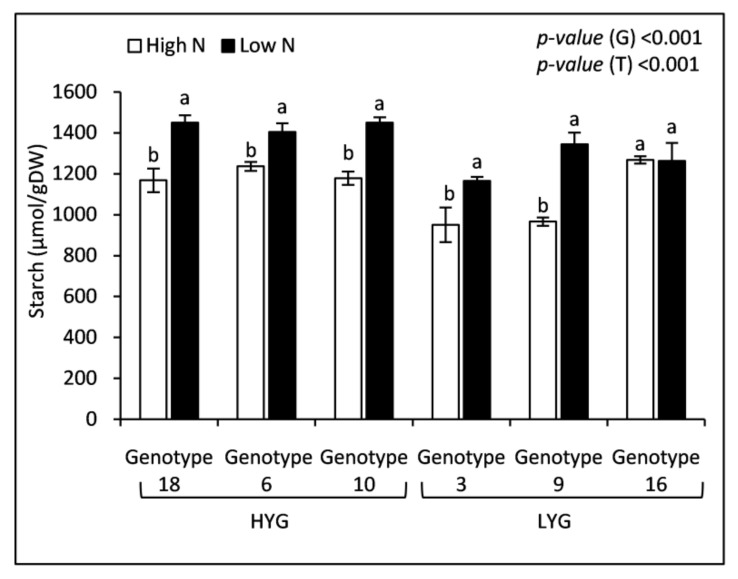
Grain starch concentration of 6 wheat genotypes fertilized by two nitrogen levels (High and Low). Data correspond to the means ± standard error (n = 4). For each genotype, letters indicate significant differences between the two treatments at *p* < 0.5. *p*-values corresponds to two-way ANOVA analysis. HYG: High-yielding genotypes, LYG: Low-yielding genotypes, G: Genotype, T: Treatment.

High N fertilization affected positively soluble sugars concentration in grain (Figure 1). Aside from fructose, the other sugars (glucose, sucrose and maltose) increased significantly (*p* < 0.001) (Figure 1). Maltose recorded the largest increase (+117.5%), followed by glucose (+60.24%), while fructose and sucrose concentrations also rose but much less (+6.65% and +4.6%, respectively) (Figure 1). Genotypes showed also significant differences for all soluble sugars (*p* < 0.001) except for maltose (*p* = 0.35) (Figure 1). It should be noted that monosaccharide concentrations (glucose and fructose) of high-yielding genotypes (18, 6 and 10) were higher when compared to those of low-yielding ones (3, 9 and 16) (Figure 1). Nevertheless, grain starch concentration declined significantly under the high N treatment for all genotypes except genotype 16 that showed similar data under the two treatments (Figure 2). The mean change in starch concentration recorded in the high N treatment was −16.03% relative to the low N one. As occurred for the monosaccharide concentrations, grain starch was higher in the high-yielding genotypes (Figure 2).

### 2.4. Wheat Grain Gliadin and Glutenin Concentrations

**Figure 3:**
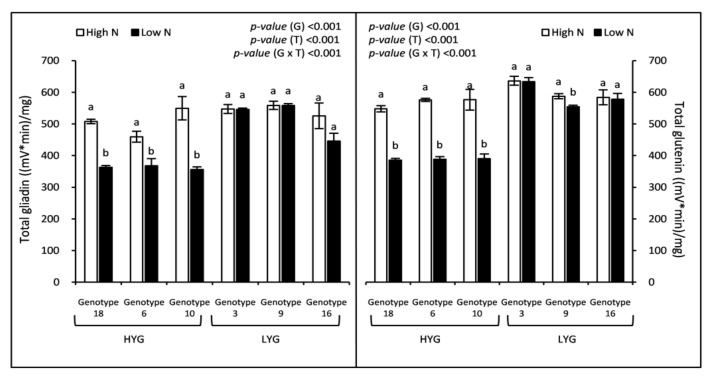
Grain gliadin and glutenin concentrations of 6 wheat genotypes fertilized by two nitrogen levels (High and Low). Data correspond to the means ± standard error (n = 4). For each genotype, letters indicate significant differences between the two treatments at *p* < 0.5. *p*-values corresponds to two-way ANOVA analysis. HYG: High-yielding genotypes, LYG: Low-yielding genotypes, G: Genotype, T: Treatment.

**Figure 4:**
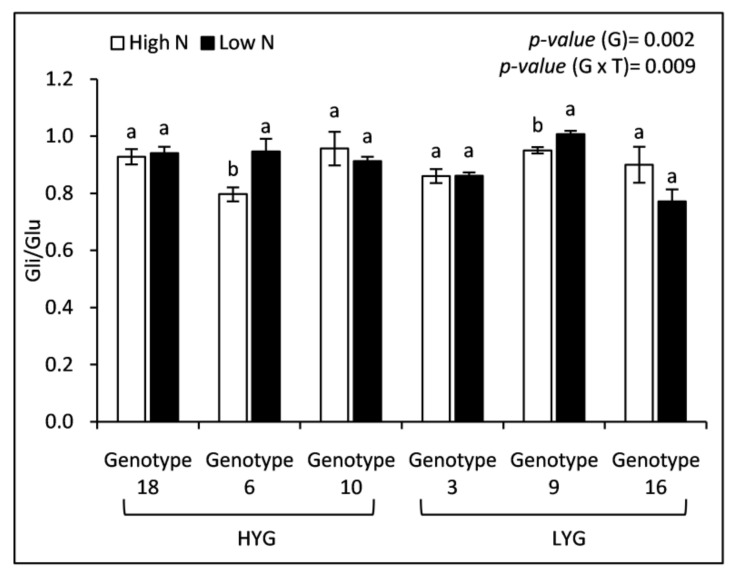
Grain gliadin to glutenin ratio (Gli/Glu) of 6 wheat genotypes fertilized by two nitrogen levels (High and Low). Data correspond to the means ± standard error (n = 4). For each genotype, letters indicate significant differences between the two treatments at *p* < 0.5. *p*-values corresponds to two-way ANOVA analysis. HYG: High-yielding genotypes, LYG: Low-yielding genotypes, G: Genotype, T: Treatment.

As expected, N supply stimulated the synthesis of gliadins and glutenins in wheat grains but the increases were genotype-dependent (Figure 3). Results presented in Figure 3 indicated that high-yielding genotypes increased significantly total gliadin and glutenin concentrations, while it remained more or less constant for low-yielding genotypes. Thus, with respect to the change in mean concentration due to the high N treatment, total gliadins and glutenins were increased by 39.73% and 46.05%, respectively, for the high-yielding genotypes, while the increases in low-yielding genotypes were insignificant (+6.07% and +2.44%). Significant differences were detected among genotypes with the highest mean values recorded for the low-yielding wheat genotypes (Figure 3). Gliadin to glutenin (Gli/Glu) ratio ranged from 0.77 to 1.01 under low N and from 0.79 to 0.95 under high N (Figure 4). A high N fertilization did not affect Gli/Glu ratio, whereas genotype did (Figure 4). Genotypes 6 and 9 showed a significant decrease in their Gli/Glu ratios under the high N treatment, however, this trait remained fairly constant in the other genotypes. The analysis of the gliadin’s fractions showed that the α/β fraction was quantitatively predominant, increasing by 18.97% under high N (Table 3). ω and γ fractions also increased significantly in response to a higher N supply, by 35.48% and 11.19%, respectively (Table 3). The differences of high- and low-yielding genotypes with respect to N fertilization described for total gliadins (Figure 3) were also observed for their fractions (Table 3). Glutenin fractions were also significantly affected when N increased (Table 3). High (HMW) and low molecular weight (LMW) subunits of glutenins significantly increased in the high N treatment when compared with the low one, being the largest increase found (23.25%) in those of HMW (Table 3).

### 2.5. Wheat Grain Mineral Composition

Grain micro- and macro-nutrients concentration was significantly increased in wheat grown under high N (Table 4). Only Zn showed a slight, non-significant increase of 1.81%. Across the 10 nutrients that increased, the mean change ranged between 3.69% and 84.77% (*p* = 0.023, *p* < 0.001). The lowest increases were recorded for Cu and K (3.69% and 4.85%, *p* = 0.033 and *p* = 0.008, respectively), whereas Ca and Al increased the most (31.44% and 84.77%, respectively, *p* < 0.001). In general, whether in high or low N level, the mean concentration of most nutrients recorded for the low-yielding genotypes (3, 9, and 16) was larger than that recorded for the high-yielding ones (18, 6 and 10) (Table 4). Among the genotypes, specifically, genotype 3 had generally the highest nutrient values both under low N as well as under high N supply (Table 4).

### 2.6. Wheat Grain Polyphenols and Anti-Radical Activity

Soluble and hydrolysable polyphenols (in mg gallic acid 100 g^−1^ flour) constitute minor (range between 172 and 297) and major (range between 525 and 928) sub-fractions, respectively, in all wheat genotypes under both N treatments (Table 5). In response to an increase in N supply, a significant decrease was observed in soluble polyphenols (−19.36%); meanwhile, hydrolysable polyphenols increased 47%. A significant difference was also detected among genotypes under both N treatments (Table 5). In fact, under high N, soluble and hydrolysable polyphenols concentrations were higher in low-yielding genotypes but, under low N, soluble polyphenols concentration was higher in high-yielding genotypes and no significant difference was detected for hydrolysable polyphenols. Bio-accessible polyphenols accounted for 44–66% and 59–74% of the total polyphenols under high and low N (Table 5), in line with a significant decrease (−4.62%) in the mean concentration of grain bio-accessible polyphenols in response to an increased N supply (Table 5). As a consequence of the already mentioned changes in soluble and hydrolysable polyphenols, total polyphenols concentration significantly increased (25.21%), the highest accumulation being observed in low-yielding genotypes (Table 5). Additionally, anti-radical activity was determined by the extent of the reduction of the stable 2,2-diphenyl-1-picrylhydrazyl (DPPH) radical. Results correspond to the remaining unreacted DPPH amount when 0.494 μmol of the free radical is initially available to react with 2.4–2.6 mg of flour extracted with methanol/acetone/water (Table 5). The comparison between the two N treatments gave similar anti-radical activities (a mean of 53%, *p* = 0.91). However, significant differences were detected among genotypes (Table 5). Aside from genotype 3, results indicate that, under high N supply, anti-radical activity of the high-yielding genotypes was higher than that of the low-yielding genotypes (genotypes 9 and 16), while an opposite trend was observed under the low N treatment.

## 3. Discussion

### 3.1. Grain and Thousand-Grain Weight Traits Were Used to Differentiate High- and Low-Yielding Durum Wheat Genotypes

In the current study, 20 genotypes from CIMMYT were chosen from a set of 120 genotypes as the ones having highest grain protein and/or starch content. Grain yield per plant and TGW traits were used to identify genotypes of contrasting grain production among 20 durum wheat genotypes tested. Genotypes 18, 6 and 10 were selected as high-yielding genotypes since they recorded the highest values of grain yield and TGW under the two N levels tested, while genotypes 3, 9 and 16 showed the lowest values and were tagged as low-yielding genotypes. These genotypes were used to investigate the effects of N fertilization in grain yield and quality.

### 3.2. A Supplementary Nitrogen Addition Post-Anthesis Slightly Improves Durum Wheat Grain Yield

Nitrogen fertilization generally [9,31,32,33] but not always [34] stimulates grain yield in triticale and wheat. Our results with durum wheat are in line with the former rather than the latter since grain yield significantly increased (7%) in the high N treatment with respect to the low N supply when applied post-anthesis with a similar behavior in all genotypes tested. The observed increase in grain yield can be attributed to many components, such as ears number, number of grains produced per ear, and TGW that may respond positively to N fertilization. In the current study, we suggest that the increase of wheat grain yield observed under the high N treatment might be due to the increase in grain number produced per plant because (i) TGW increased, but not significantly (1.3%), under the high N treatment with respect to the low one, and (ii) no significant differences between treatments were found in a fast screening made for number of tillers per plant (not shown). These results agree with those reported by Li et al. [35] and Abedi et al. [17] in bread wheat.

### 3.3. A High Nitrogen Supply Increases Durum Wheat Grain Nitrogen but Decreases Nitrogen Use Efficiency

We fertilized plants from anthesis to maturity. The recommendation of applying N several times to the durum wheat crop, in order to achieve greater efficiency, is commonly accepted. However, the number of applications may vary. Thus, in some cases, the proposed fertilization includes application during sowing and vegetative growth up to flowering phase [36,37]. However, it has been shown that a late supply of N increases the protein content of the grain and improves the quality of durum wheat [38]. In the experimental design of our experiment, special emphasis was made on studying the ability of the different genotypes to use this late application of N, considering that the N present in the initial substrate covered the needs of the crop up to anthesis in an analogous way to the applications that farmers usually carry out during sowing and pre-anthesis. Our approach of using pots and greenhouse, however, simplify the logistics (field surface to be cultivated, number of analyses, etc.) and can be used as a pre-selection trial for data based genotype choice to be planted in a field test.

Using the above-mentioned approach, N concentration was, as expected, increased in durum wheat grains regardless of yields when the N dose was increased. This could be due to a large N availability post-anthesis coming from root N uptake and N remobilization to the grain during grain filling [39]. Belete et al. [39] and López-Bellido et al. [40] found genotypic variability in grain N content at different N rates between genotypes of high and low yield. In line to that, the highest amount of grain N was recorded in the low-yielding genotypes irrespective of N supply. The low N concentration in grains of the high-yielding genotypes when grown at low N could be explained by dilution of N due to higher yields comparing them to those of the low-yielding genotypes. On the other hand, C grain concentration also significantly increased under the high N treatment but the extent of the increase was small, much lower than that of the grain N increase (+2.43% vs. +29.24%), which explains the decrease in C/N ratio. In rice panicle at filling and maturity stages Ye et al. [41] and mature wheat grains Yan et al. [42] found similar results. In this line, it has been reported that tissue C concentration is relatively unaffected by N fertilization due to the stable plant C structural basis that accounts almost for 50% of plant dry mass [41,42]. Nitrogen use efficiency (NUE) was highly influenced by the applied N dose and genotype. An increased N fertilization rate led to a decrease in NUE. This is coherent because a large part of the additional N is driven to grain protein synthesis whereas yield increases were small (i.e., the concentration of N in the grain increases more than the yield). This result confirms previous reports made working with bread [33,39,43] and durum [44] wheat. Under both N treatments, high-yielding genotypes showed higher efficiency in the use of the applied N. This finding led us to hypothesize that NUE can be a trait under genetic control. Having highest or lowest NUE between genotypes was independent of N dose, which is an advantage when using this parameter in genetic improvement programs, although it is true that under conditions of low N is where the differences were most marked. Thus, broader germplasm screening for varieties with high NUE under limited N resources using pots and facilities, such as greenhouses, could be an easier, useful tool to select genotypes aimed to improve durum wheat crop yield and minimize environment contamination due to excessive N fertilization in field-based trials.

### 3.4. Durum Wheat Grain Carbohydrates Tend to Be Stored as Mono and Disaccharides (Glucose, Sucrose, and Maltose), Not as Starch, When the Applied N Is High

Durum wheat grain non-structural carbohydrates concentrations were highly dependent on N dose and genotype. Soluble sugars (glucose, maltose, and sucrose) were more abundant in grain at high than at low N application. On the contrary, starch concentration decreased with increasing N availability. It can be concluded that, under the high N treatment, grain carbohydrates tend to be stored as mono and disaccharides (glucose, sucrose, and maltose) not as starch, suggesting a slowed down starch synthesis. Results from different crops agree with our findings. Zadeh et al. [45] reported that starch in rice could be increased due to a moderate reduction in N. Starch in developing and mature maize kernel was negatively correlated with N availability, whereas the response of glucose and fructose in developing maize ears toward N supply was opposite to starch [46]. Similarly, Galani et al. [47] reported that N fertilizer increased soluble carbohydrates, mainly sucrose, in sweet sorghum. Matching results were also presented by Almodares et al. [48] and Asthir et al. [6] in sweet sorghum and wheat, respectively. Taken together, all these findings suggest that high N dose stimulates soluble sugars biosynthesis and reduces their conversion into starch. It has been reported that, N fertilization increases the triose-phosphate/phosphate translocation activity, as well as sucrose-phosphate synthase1 (*ZmSps1*), leading to more C flux to sucrose synthesis than to starch accumulation in maize leaves [46]. Polysaccharides, hexoses, and disaccharides concentrations in durum wheat grain were higher in high-yielding genotypes under both N doses. It can be hypothesized a higher photosynthetic capacity of their leaves and C fixation into carbohydrates in those genotypes.

Regarding the technological quality of durum wheat, there is no evidence that an increase in the content of soluble sugars affects its aptitude for making pasta. On the other hand, it could have a negative effect on how “healthy” the resulting pasta is, since increasing the soluble sugars increases the glycemic index. It must be taken into account that pasta is considered a food with a low glycemic index [49].

### 3.5. N Supply Stimulated the Synthesis of Storage Proteins, Gliadins and Glutenins, in Durum Wheat Grains but Only in the High-Yielding Genotypes

As above discussed, a higher N supply resulted in an accumulation of N in the durum wheat grain, putatively in form of grain protein. Gliadins and glutenins are storage proteins with the latter being more important than gliadins for obtaining good dough properties [50,51,52]. In line with previous reports [16,53], our results showed that not only gluten concentration is increased but also its composition is impacted by N fertilization. Although the 3 fractions of gliadins increased, the highest N-mediated increase was observed in ω-gliadins. In the case of glutenin fractions, both HMW and LMW increased with a higher N dose being the increase a little bit higher for the HMW fraction. In bread wheat, this greater increase in the synthesis of ω-gliadins and HMW glutenins has been related to the proportion of amino acids that contain S (Cys and Met) in both types of proteins [54]. An interesting output of our work is that protein accumulation during grain filling seems to have also a genetic component. Low-yielding genotypes showed the highest concentrations of gliadins and glutenins (and consequently of their fractions) under both treatments; however, high-yielding genotypes appear to be more sensitive to N supply (raising N dose does not affect gluten content in grains of low-yielding plants). Under low N dose, the accumulation of carbohydrates in high-yielding genotypes was much larger than that observed in the low-yielding ones, which can explain the low concentration of gliadins and glutenins in their grains. The larger gluten content found in high-yielding genotypes grown under high N fertilization in the current work should be related to an increased N uptake and/or N remobilization to grain in these genotypes. In addition, since it is a relatively late N contribution (post-anthesis), the ability of the plant to use that N to produce more grain is limited, using it to synthesize reserve proteins. Gliadin to glutenin ratio was not affected by the N treatment when considered all genotypes as a whole (although the ratio decreased at high N in genotypes 6 and 9), which indicates that this trait is controlled genetically. These results agree with those reported by Johansson et al. [55], who found that this ratio was only influenced by the cultivar. In the literature, there are examples of increases [56], decreases [57], and no changes [58,59] of the gliadin to glutenin ratio in response to high N.

### 3.6. A High Nitrogen Supply Increases Durum Wheat Grain Mineral Concentrations

From the literature, a clear conclusion on how N supply affects macro- and micro-nutrients concentrations in wheat grain cannot be obtained. On one hand, there are reports showing that late N application at heading increased macro- and micro-nutrients (Zn, Fe, and Mg) grain concentrations [24,60]. On the other hand, Dolijanovic et al. [7] concluded that a reduced N application (60 vs. 120 kg ha^−1^) had a positive effect on the concentration of nutritionally important minerals (Ca, Cu, Fe, K, Mg, Mn, P, and Zn). In line with this, Smith et al. [26] reported that grain concentration of P, K, Ca, Mg, Mn, and Zn declined as crop yields increased in response to N fertilization. These latter results appear to be contradictory with the fact that, according to our results, increasing the N dose post-anthesis enhances slightly grain yield and does raise grain mineral concentrations for both high- and low-yielding genotypes. Causes for the enhanced grain mineral concentrations under higher N supply have been ascribed to a higher root growth that promotes nutrient uptake [23,60]. In bread wheat, grain mineral concentrations tend to decrease as yields increase; therefore, breeding for yield improvement may reduce wheat nutritional quality [61]. When comparing among genotypes, it should be highlighted that there was a large variability with respect to grain nutrients, which indicates that grain mineral composition is in part controlled genetically. Low-yielding genotypes had the highest mineral concentrations (K, P, Mg, S, Na, Ca, Mn, Fe, Zn, and Cu) under both N treatments, suggesting a “concentration” effect. In addition, the lower values of grain nutrients that had the high-yielding genotypes can be related to a “dilution” effect due to their higher yields regardless of the N applied. Finally, other reports have shown no changes with N supply in most nutrients analyzed (P, K, Mg, and Na), only Ca increased [62]. Our results with N fertilization from anthesis to maturity are in line with those of Zhao et al. [63] investigating water availability at post-anthesis. These authors found that the concentration of Zn and Fe has a significant positive correlation with grain P and protein.

### 3.7. A High Nitrogen Supply Increases Total Grain Polyphenols, Due to the Hydrolysable Fraction, but with No Impact on the Anti-Radical Activity

Few studies have analyzed the effect of N fertilization on polyphenols in grains, with their results being divergent. Engert et al. [2] and Ma et al. [28] found that total phenolics increased in wheat grains due to N fertilization, while N availability affected negatively total phenolics in tef grains [64] and sesame seeds [65]. Stumpf et al. [66] found, on the contrary, that total phenolic concentration was not affected by N treatment in wheat grains. These differences could be explained by the different amounts of N fertilizer and/or the genotype used for the experiments. Our results agree with the two former of these studies. In response to N fertilization increase, grain polyphenols concentration and composition were significantly modified. A clear interaction in soluble and bio-accessible polyphenols was observed, i.e., decrease in soluble polyphenols in high-yielding genotypes with no change in the low-yielding ones, and decrease in bio-accessible polyphenols in low-yielding genotypes compared to a slight increase in the high-yield ones. When analyzing the variation of polyphenol fractions in response to a rise in the N fertilization dose, hydrolysable polyphenols explain, to a large extent, the augmentation in grain total polyphenols. Our results on soluble polyphenols contrast with those of Stumpf et al. [66] and Langenkämper et al. [67], who reported that soluble phenolics were higher in grains of unfertilized wheat compared to fertilized plants. The increase in total polyphenols could be ascribed to the sufficient availability of phenylalanine under high N application. This amino acid is a key metabolite in the synthesis of phenols, together with its use in the route for protein biosynthesis [68]. In soybean, N fertilization has been related with phyto-hormones production and phenolics as growth-promoting compounds [69]. Ma et al. [28] suggested that the antioxidant activity in wheat grain could be increased with an adequate N application. Our data indicate that fertilization had no effect on the anti-radical activity when considering all genotypes as a whole, in agreement with previous reports [66,70]. However, there was a genotype x treatment interaction. At low N, the low-yielding genotypes had more anti-radical activity, but, with high N, it was the other way around.

## 4. Materials and Methods

### 4.1. Plant Material and Experimental Design

The experiment was carried out from March to July 2017 with 20 durum wheat (*Triticum turgidum* L. var. *durum*) genotypes. Seeds were obtained from CIMMYT (International Maize and Wheat Improvement Center, Mexico) (Table S1). Those genotypes were selected from a set of 120 genotypes as the ones having highest grain protein and/or starch content. Seedlings were vernalized for one week in a cold room at 4 °C and then transplanted to 6 L pots containing a peat/perlite/vermiculite 2:2:1 (*v*/*v*/*v*) substrate mixture. After sowing, the plants were transferred to a greenhouse located at the Institute of Agrobiotechnology (IdAB), Pamplona (Spain). Plants were grown under natural sunlight (with no supplemental lighting) and day-length at 15–17/19–23 °C from March to June and 17–20/19–26 °C in July night/day. Plants were irrigated with water until they reached anthesis stage (nutrients were released from peat). Then, half of the plants were watered by ½ Hoagland nutrient solution (100% N: high N treatment), whereas the other half were watered with modified ½ Hoagland solution with 1/3 N (low N treatment). For each treatment, 4 pots were used per genotype, with 2 plants per pot, and the experiment was conducted according to randomized complete block design. Plants were irrigated 3 times per week (0.5 L each per pot), two times with Hoagland solution and once with water. At maturity, plants were harvested, and grains were collected, to determine yield and quality traits of the different genotypes.

### 4.2. Grain Yield and Thousand-Grain Weight

Grain yield and thousand-grain weight (TGW) were determined for each plant. TGW was determined by calculating the weight of 20 grains and then converted to the weight of 1000 grains as follows: TGW(g) = (weight of 20 grains (g) × 1000)/20. These parameters were determined for the 20 genotypes, as a prerequisite to select 6 genotypes (3 as high-yielding genotypes and 3 as low-yielding ones) in order to analyze grain quality under the two above-mentioned N levels.

### 4.3. Grain Carbon and Nitrogen Concentrations and Nitrogen Use Efficiency (NUE)

C and N concentration (% of dry weight (DW)) analyses were determined using an elemental analyzer (FlashEA1112, ThermoFinnigan, Waltham, MA, USA) equipped with a MAS200R autosampler. Grains were ground to a fine powder and ≈1 mg samples were weighed and stored in tin capsules for elemental analyses (MX5 microbalance, Mettler-Toledo, Columbus, OH, USA) and introduced into a quartz reactor filled with WO_3_ and copper and heated at 1020 °C. The combustion gas mixture was carried by a helium flow to a WO_3_ layer to achieve a complete quantitative oxidation, followed by a reduction step in a copper layer to reduce nitrogen oxides and SO_3_ to N_2_ and SO_2_. The resulting components, N_2_, CO_2_, H_2_O, and SO_2_ were separated in a chromatographic column (Porapak 2m, Santa Clara, CA, USA) and detected with a thermal conductivity detector. Nitrogen use efficiency (NUE) of production was determined as the ratio of grain yield to the total N concentration in grains, which must be differentiated from other ways of measuring the efficiency of N use by plants [71].

### 4.4. Grain Mineral Composition

Micro- and macro-nutrients concentrations were determined by inductively coupled plasma/optical emission spectrometry (ICP/OES, iCAP 6500 Duo, Thermo Fisher Scientific, Waltham, MA, USA).

### 4.5. Grain Carbohydrates Composition

Wheat grains were milled, and 25 mg of each sample were added to 0.5 mL of 100% ethanol then another 0.5 mL of 80% ethanol was added and heated in a thermomixer (70 °C, 90 min, 1100 rpm). The mixture was centrifuged (22 °C, 10 min, 20,800× *g*) and the supernatant was used for the determination of soluble sugars (glucose, fructose and sucrose) concentration, using an ionic chromatographer (ICS-3000, Thermo ScientificTM, Waltham, MA, USA). Reference was made to sugar standards of known concentrations (50 mM). The pellet was used to determine starch content. Starch was solubilized by adding potassium hydroxide (KOH) (0.2 N) to the pellet, and the pH was adjusted to 4.8 with acetic acid (0.1 N). Quantification was performed with the kit containing the enzyme amyloglucosidase (R-Biopharm, AG; Darmstadt, Germany) measuring the absorbance at 340 nm with a spectrophotometer.

### 4.6. Grain Gliadin and Glutenin Concentrations

To determine grain gliadin concentration, 167 mg of milled samples were placed in 2 mL test tube. First, albumins and globulins were extracted with 1 mL of buffer A (0.05 M sodium phosphate pH 7.8 and 0.05 M NaCl) for 1 h at 4 °C. Extraction was followed by centrifugation at 20,800× *g* for 5 min at 4 °C. Amphiphilic proteins were then extracted from the pellet resuspended in 1 mL of 2% (*w*/*v*) Triton X-114 in buffer A for 1 h at 4 °C. After centrifugation at 20,800× *g* for 5 min at 4 °C, gliadins were separated from the residue with 1 mL of 70% (*v*/*v*) aqueous ethanol for 1 h at 20 °C and centrifuged at 20,800× *g* for 5 min at 20 °C. Gliadins were located in the supernatant. For glutenin extraction, only 50 mg of white flour were used to avoid a too-viscous supernatant after glutenin extraction. After gliadin extraction, glutenins were extracted overnight with 1.5 mL of buffer B (0.05 M disodium tetraborate pH 8.5, 2% (*v*/*v*) β-mercaptoethanol, 8 M urea, and 1 g L^−1^ glycine) at 20 °C. Samples were centrifuged at 20,800× *g* for 5 min at 20 °C, and an aliquot of 0.5 mL was alkylated with 15 μL of 4-vinylpyridine for 45 min at 60 °C. Afterward, 1 volume of 2-propanol was added to precipitate polysaccharides, and samples were centrifuged at 20,800× *g* for 1 min at 20 °C. Supernatants containing gliadins and glutenins were filtered with a 0.45 μm polyvinylidene fluoride filter before quantification by reverse phase high performance liquid chromatography (RP-HPLC) in a Waters 2695 Separations Module (Waters Corporation, Milford, MA, USA) using a Europa Protein 300 C18 column (300 Å, 5 μm, and 250 × 4.6 mm) at 50 °C with a Guard Column Protein 300 C18 (10 × 3.2 mm) (Teknokroma, Sant Cugat del Vallès, Spain). Eluents and gradient conditions for RP-HPLC were as described in Triboi et al. [72]. Amounts of gliadins and glutenins were calculated by integration of the areas under the curve of the ultraviolet signal (220 nm) and expressed as the chromatogram area per milligram of whole-meal flour on a dry matter basis (mV min mg^−1^).

### 4.7. Grain Polyphenol Fractions and Anti-Radical Activity

Soluble polyphenols were determined in 500 mg of freeze ground grain samples by extraction with 4 mL of acidified methanol (HCl/methanol/water, 1:80:10, *v*/*v*/*v*) at room temperature for 2 h [73]. Hydrolysable polyphenols were determined in sample residues remained after extraction of soluble phenolics, by mixing with 5 mL of methanol and concentrated sulfuric acid (10:1, *v*/*v*) at 85 °C for 20 h in a shaking water bath [74]. Bio-accessible polyphenols were assessed by conducting an “in vitro” digestive enzymatic mild extraction that mimics the conditions in the gastrointestinal tract, according to the procedure of Glahn et al. [75] and adapted for flours and breads by Angioloni and Collar [76]. Supernatants obtained after extractions were combined and used for respective determination of soluble, hydrolysable, and bio-accessible polyphenols using the Folin-Ciocalteu spectrophotometric method. A calibration curve was made using gallic acid; therefore, obtained amounts of phenolics were expressed as gallic acid equivalents. The stable 2,2-diphenyl-1-picrylhydrazyl (DPPH) radical was used to measure the radical scavenging capacity of milled samples according to the DPPH method modified by Sánchez-Moreno et al. [77] and adapted by Collar et al. [78]. Plots of micromoles of DPPH versus time (min) were drawn and calculations were made to determine the anti-radical activity (AR) as follows: ((DPPH INITIAL − DPPH PLATEAU) × 100)/DPPH INITIAL.

### 4.8. Statistical Analysis

To explore the N fertilization effect on yield components and used to select 6 out of the 20 genotypes tested, one-way analysis of variance (ANOVA) (Statgraphics, Centurion XV, Version 15.1.02, Madrid, Spain) was conducted using ‘genotypes’ as factor. Two-way analysis of variance has been also conducted to analyze the interaction between genotypes and N treatments. Regarding to grain quality parameters, univariate statistic analyses were performed by two-way analysis of variance using the factor ‘N treatments’ with two values (high and low) and the factor ‘genotypes’ having 6 values (6 different genotypes). One-way ANOVA was also performed to evaluate differences among genotypes. Results were considered to be significant when *p* < 0.05. When the main factors ‘genotypes’ or ‘N treatments’ gave statistically significant differences, least significant difference (LSD) was used to determine statistical differences among genotypes and treatments.

## 5. Conclusions

Nitrogen fertilization applied from anthesis to maturity had small effects on durum wheat grain yield but had a major impact on grain quality. Traits, such as grain yield and TGW, were used to differentiate high- and low-yielding genotypes. A higher N supply increased grain N concentration but decreased NUE. A higher N availability during grain filling resulted in an overall enhancement in nutritional grain quality. Storage proteins, gliadin and glutenin, soluble sugars, minerals, and phenolic compounds were increased in mature grains. Results on grain yield and quality were genotype-dependent, particularly characteristically different in high- and low-yielding genotypes. A crop, like durum wheat, has quality requirements that are based, in part, on a high protein content in the grain. To achieve this protein content, an extra supply of N will be needed, which will inevitably reduce the NUE, but it cannot be considered as intrinsically negative. Therefore, to meet the future demands of global population, screening for genotypes with an adequate balance between high, stable yields, and satisfactory nutritional values and NUE could be a suitable alternative.

## Figures and Tables

**Table 1:** Grain yield and thousand-grain weight (TGW) of 20 wheat genotypes fertilized by two nitrogen levels (High and Low). Means are followed by standard error (n = 8).

Genotypes	Grain Yield (g/Plant)		TGW (g)	
	High N	Low N	High N	Low N
18	9.43 ^a^ ± 0.58	8.22 ^ab^ ± 0.28	58.63 ^a^ ± 1.88	56.50 ^a^ ± 2.49
4	9.09 ^ab^ ± 0.57	6.57 ^defg^ ± 0.49	50.75 ^cd^ ± 1.69	47.14 ^e^ ± 0.61
6	8.68 ^abc^ ± 0.46	7.85 ^abc^ ± 0.54	57.25 ^ab^ ± 0.54	51.38 ^cd^ ± 2.43
5	8.40 ^abcd^ ± 0.45	6.94 ^cdef^ ± 0.52	48.75 ^de^ ± 0.53	47.69 ^de^ ± 1.23
8	8.33 ^abcd^ ± 0.48	7.72 ^abcd^ ± 0.47	51.06 ^cd^ ± 1.02	52.50 ^bc^ ± 1.05
15	7.98 ^abcdef^ ± 0.82	8.27 ^a^ ± 0.50	51.00 ^cd^ ± 1.67	51.81 ^bc^ ± 0.91
14	7.96 ^bcde^ ± 0.42	6.92 ^cdef^ ± 0.34	46.56 ^efg^ ± 1.34	48.94 ^cde^ ± 1.52
10	7.41 ^cdefg^ ± 0.31	7.36 ^abcdef^ ± 0.39	52.88 ^c^ ± 1.00	52.13 ^bc^ ± 1.42
1	7.31 ^cdefg^ ± 0.34	6.37 ^efgh^ ± 0.55	46.38 ^efg^ ± 1.41	42.88 ^fg^ ± 1.83
13	7.17 ^defg^ ± 0.36	7.55 ^abcde^ ± 0.45	53.50 ^bc^ ± 0.73	50.00 ^cde^ ± 1.33
2	7.16 ^defg^ ± 0.39	6.90 ^cdef^ ± 0.40	43.56 ^g^ ± 1.13	42.44 ^g^ ± 1.45
12	7.06 ^defgh^ ± 0.45	6.23 ^fgh^ ± 0.45	54.25 ^bc^ ± 1.88	50.38 ^cde^ ± 1.22
19	6.86 ^efgh^ ± 0.52	7.05 ^bcdef^ ± 0.38	43.56 ^g^ ± 1.02	50.63 ^cde^ ± 0.69
7	6.83 ^efgh^ ± 0.75	5.19 ^h^ ± 0.35	43.94 ^fg^ ± 1.52	52.06 ^bc^ ± 0.65
11	6.78 ^efgh^ ± 0.64	6.52 ^efg^ ± 0.44	58.56 ^a^ ± 1.16	55.69 ^ab^ ± 1.57
20	6.70 ^efgh^ ± 0.30	7.77 ^abcd^ ± 0.33	47.56 ^def^ ± 1.48	50.38 ^cde^ ± 0.91
3	6.53 ^efgh^ ± 0.37	6.28 ^fgh^ ± 0.49	44.06 ^fg^ ± 1.59	42.13 ^g^ ± 1.26
16	6.43 ^fgh^ ± 0.53	6.17 ^fgh^ ± 0.43	50.69 ^cd^ ± 0.87	46.69 ^ef^ ± 0.97
9	6.27 ^gh^ ± 0.53	5.40 ^gh^ ± 0.34	48.81 ^de^ ± 1.98	49.19 ^cde^ ± 0.64
17	5.74 ^h^ ± 0.26	7.37 ^abcdef^ ± 0.38	54.19 ^bc^ ± 2.22	52.88 ^abc^ ± 1.97
*Two-way ANOVA*				
*p*-value (G effect)	**<0.001**		**<0.001**	
*p*-value (T effect)	**0.03**		0.34	
*p*-value (G × T)	0.75		0.40	

Grey shading indicates the genotypes that were selected for grain quality analyses. Within each treatment, letters indicate significance differences between genotypes at *p* < 0.5. Values in bold indicate significance (*p* < 0.05). G: Genotype, T: Treatment.

**Table 2:** Grain nitrogen concentration (N), carbon concentration (C), C/N ratio, and nitrogen use efficiency (NUE) of 6 wheat genotypes fertilized by two nitrogen levels (High and Low). Means are followed by standard error (n = 8).

Genotypes	N (%)		C (%)		C/N		NUE	
	High N	Low N	High N	Low N	High N	Low N	High N	Low N
Genotype 18 (HYG)	2.8 ^d^ ± 0.07	2.2 ^b^ ± 0.08	41.0 ^cd^ ± 0.11	39.7 ^c^ ± 0.09	14.6 ^a^ ± 0.34	18.1 ^a^ ± 0.64	35.70 ^a^ ± 0.92	45.55 ^a^ ± 1.56
Genotype 6 (HYG)	2.9 ^cd^ ± 0.04	2.1 ^b^ ± 0.06	40.9 ^d^ ± 0.08	40.5 ^b^ ± 0.06	13.9 ^ab^ ± 0.17	19.4 ^a^ ± 0.51	34.03 ^ab^ ± 0.45	47.98 ^a^ ± 1.27
Genotype 10 (HYG)	3.1 ^bc^ ± 0.09	2.1 ^b^ ± 0.04	41.6 ^b^ ± 0.06	41.2 ^a^ ± 0.07	13.6 ^b^ ± 0.38	19.5 ^a^ ± 0.37	32.69 ^bc^ ± 0.88	47.23 ^a^ ± 0.88
Genotype 3 (LYG)	3.2 ^ab^ ± 0.12	2.6 ^a^ ± 0.13	41.3 ^c^ ± 0.11	40.7 ^b^ ± 0.14	13.1 ^bc^ ± 0.48	15.9 ^b^ ± 0.71	31.77 ^bcd^ ± 1.20	39.19 ^b^ ± 1.84
Genotype 9 (LYG)	3.4 ^a^ ± 0.10	2.7 ^a^ ± 0.10	41.2 ^c^ ± 0.06	40.3 ^b^ ± 0.15	12.2 ^c^ ± 0.36	14.8 ^b^ ± 0.55	29.52 ^d^ ± 0.87	36.78 ^b^ ± 1.44
Genotype 16 (LYG)	3.2 ^ab^ ± 0.06	2.7 ^a^ ± 0.14	42.6 ^a^ ± 0.09	40.3 ^b^ ± 0.30	13.2 ^b^ ± 0.24	15.5 ^b^ ± 0.80	31.05 ^cd^ ± 0.57	38.40 ^b^ ± 2.00
N effect (%)	+29.24 ***		+2.43 ***		−21.89 ***		−23.66 ***	
*Two-way ANOVA*								
*p*-value (G effect)	**<0.001**		**<0.001**		**<0.001**		**<0.001**	
*p*-value (T effect)	**<0.001**		**<0.001**		**<0.001**		**<0.001**	
*p*-value (G × T)	0.194		**<0.001**		**<0.001**		**0.005**	

Within each treatment, letters indicate significant differences between genotypes at *p* < 0.5. *** indicates significance at *p* < 0.001. Values in bold indicate significance (*p* < 0.05). HYG: High-yielding genotypes (grey shadowed), LYG: Low-yielding genotypes, G: Genotype, T: Treatment.

**Table 3:** Total gliadin and glutenin fractions of 6 wheat genotypes fertilized by two nitrogen levels (High and Low). Means are followed by standard error (n = 4).

N Level	Genotypes	Total Gliadin ((mv*min)/mg)			Total Glutenin ((mv*min)/mg)	
		ω	α-β	γ	HMW	LMW
High N	Genotype 18 (HYG)	25.32 ^ab^ ± 2.14	302.07 ^ab^ ± 8.59	180.31 ^cd^ ± 5.71	89.42 ^b^ ± 3.1	458.55 ^c^ ± 8.59
	Genotype 6 (HYG)	19.00 ^b^ ± 1.17	248.07 ^c^ ± 12.63	192.42 ^c^ ± 3.49	101.72 ^ab^ ± 2.98	474.39 ^bc^ ± 2.45
	Genotype 10 (HYG)	26.44 ^a^ ± 2.28	294.67 ^ab^ ± 24.52	222.74 ^ab^ ± 10.31	110.82 ^a^ ± 7.96	465.72 ^bc^ ± 13.57
	Genotype 3 (LYG)	25.57 ^a^ ± 1.65	321.29 ^a^ ± 11.17	200.51 ^bc^ ± 5.9	105.30 ^ab^ ± 2.94	522.25 ^a^ ± 12.28
	Genotype 9 (LYG)	19.54 ^b^ ± 2.22	261.51 ^bc^ ± 15.67	246.03 ^a^ ± 9.43	89.37 ^b^ ± 7.03	498.18 ^ab^ ± 1.50
	Genotype 16 (LYG)	24.80 ^ab^ ± 2.67	310.00 ^a^ ± 14.58	165.66 ^d^ ± 10.92	106.50 ^a^ ± 6.22	475.62 ^bc^ ± 20.17
Low N	Genotype 18 (HYG)	11.36 ^c^ ± 0.23	198.23 ^c^ ± 3.06	153.13 ^cd^ ± 2.36	51.46 ^c^ ± 2.18	334.15 ^c^ ± 3.47
	Genotype 6 (HYG)	12.36 ^c^ ± 1	204.05 ^c^ ± 14.89	151.81 ^d^ ± 7.13	51.53 ^c^ ± 1.94	336.48 ^c^ ± 6.84
	Genotype 10 (HYG)	13.11 ^c^ ± 0.56	180.28 ^c^ ± 4.64	162.68 ^cd^ ± 3.64	55.14 ^c^ ± 3.19	335.60 ^c^ ± 10.96
	Genotype 3 (LYG)	25.77 ^a^ ± 0.26	316.52 ^a^ ± 1.73	215.58 ^b^ ± 0.62	113.02 ^a^ ± 0.65	520.89 ^a^ ± 11.73
	Genotype 9 (LYG)	24.13 ^a^ ± 0.73	303.38 ^a^ ± 6.13	237.23 ^a^ ± 2.21	101.13 ^b^ ± 2.08	453.27 ^b^ ± 3.41
	Genotype 16 (LYG)	17.12 ^b^ ± 2.82	258.01 ^b^ ± 14.87	165.69 ^c^ ± 6.99	117.08 ^a^ ± 7.23	462.10 ^b^ ± 12.67
N effect (%)		+35.48 ***	+18.97 ***	+11.19 ***	+23.25 ***	+18.51 ***
*Two-way ANOVA*						
*p*-value (G effect)		**0.002**	**<0.001**	**<0.001**	**<0.001**	**<0.001**
*p*-value (T effect)		**<0.001**	**<0.001**	**<0.001**	**<0.001**	**<0.001**
*p*-value (G × T)		**<0.001**	**<0.001**	**<0.001**	**<0.001**	**<0.001**

Within each treatment, letters indicate significant differences between genotypes at *p* < 0.5. *** indicates significance at *p* < 0.001. Values in bold indicate significance (*p* < 0.05). HYG: High-yielding genotypes (grey shadowed), LYG: Low-yielding genotypes. HMW: High molecular weight, LMW: Low molecular weight, G: Genotype, T: Treatment.

**Table 4:** Grain micro- and macro-nutrients of 6 wheat genotypes fertilized by two nitrogen levels (High and Low). Means are followed by standard error (n = 4).

N Level	Genotypes	K (mg/100 g)	P (mg/100 g)	Mg (mg/100 g)	S (mg/100 g)	Na (mg/100 g)	Ca (mg/100 g)	Mn (mg/100 g)	Fe (mg/100 g)	Zn (mg/100 g)	Cu (mg/100 g)	Al (mg/100 g)
High N	Genotype 18 (HYG)	513.82 ^bc^ ± 30.39	367.42 ^bc^ ± 21.03	113.76 ^b^ ± 6.85	99.25 ^b^ ± 5.45	40.04 ^b^ ± 1.71	20.45 ^d^ ± 0.94	4.91 ^c^ ± 0.05	2.24 ^c^ ± 0.11	1.56 ^e^ ± 0.02	0.44 ^d^ ± 0.03	0.40 ^b^ ± 0.07
	Genotype 6 (HYG)	504.41 ^c^ ± 19.93	353.91 ^c^ ± 13.44	112.85 ^b^ ± 4.40	110.96 ^ab^ ± 6.96	39.23 ^b^ ± 2.24	25.07 ^c^ ± 0.86	5.26 ^bc^ ± 0.22	2.65 ^b^ ± 0.07	1.79 ^d^ ± 0.03	0.53 ^b^ ± 0.02	1.18 ^a^ ± 0.09
	Genotype 10 (HYG)	554.13 ^ab^ ± 5.96	404.93 ^b^ ± 4.18	128.86 ^a^ ± 1.24	109.13 ^ab^ ± 0.89	51.62 ^a^ ± 2.74	24.76 ^c^ ± 1.18	5.44 ^ab^ ± 0.04	2.43 ^bc^ ± 0.05	1.61 ^e^ ± 0.06	0.46 ^cd^ ± 0.00	0.55 ^b^ ± 0.13
	Genotype 3 (LYG)	595.62 ^a^ ± 9.73	442.43 ^a^ ± 8.02	136.54 ^a^ ± 2.06	114.96 ^a^ ± 1.90	51.38 ^a^ ± 1.90	30.25 ^b^ ± 0.59	5.76 ^a^ ± 0.08	3.35 ^a^ ± 0.20	2.55 ^a^ ± 0.05	0.61 ^a^ ± 0.01	0.58 ^b^ ± 0.04
	Genotype 9 (LYG)	553.28 ^ab^ ± 9.67	402.62 ^b^ ± 6.81	139.21 ^a^ ± 2.51	116.79 ^a^ ± 1.64	53.55 ^a^ ± 2.90	34.82 ^a^ ± 0.95	5.42 ^b^ ± 0.07	3.26 ^a^ ± 0.09	2.24 ^b^ ± 0.02	0.54 ^b^ ± 0.01	0.99 ^a^ ± 0.06
	Genotype 16 (LYG)	552.34 ^ab^ ± 5.97	405.25 ^b^ ± 4.21	129.55 ^a^ ± 1.12	100.62 ^b^ ± 1.43	43.32 ^b^ ± 0.73	30.94 ^b^ ± 0.73	5.53 ^ab^ ± 0.07	3.15 ^a^ ± 0.18	2.11 ^c^ ± 0.04	0.50 ^bc^ ± 0.01	0.32 ^b^ ± 0.08
Low N	Genotype 18 (HYG)	549.15 ^a^ ± 4.64	354.80 ^bc^ ± 2.17	102.10 ^b^ ± 0.51	85.46 ^b^ ± 0.76	36.63 ^b^ ± 2.35	19.12 ^cd^ ± 0.47	4.81 ^bc^ ± 0.04	2.78 ^ab^ ± 0.24	1.91 ^bc^ ± 0.03	0.46 ^b^ ± 0.00	0.40 ^b^ ± 0.05
	Genotype 6 (HYG)	563.80 ^a^ ± 24.31	345.24 ^c^ ± 14.11	105.69 ^b^ ± 4.45	88.59 ^b^ ± 3.80	38.44 ^b^ ± 0.82	19.06 ^cd^ ± 1.25	4.81 ^bc^ ± 0.20	2.32 ^b^ ± 0.08	1.85 ^c^ ± 0.10	0.53 ^a^ ± 0.02	0.73 ^a^ ± 0.09
	Genotype 10 (HYG)	503.84 ^b^ ± 3.03	356.51 ^bc^ ± 1.98	103.05 ^b^ ± 0.91	88.43 ^b^ ± 0.56	40.83 ^ab^ ± 0.28	16.16 ^d^ ± 0.18	4.56 ^c^ ± 0.05	2.64 ^ab^ ± 0.23	1.55 ^d^ ± 0.02	0.45 ^bc^ ± 0.01	0.39 ^bc^ ± 0.00
	Genotype 3 (LYG)	567.74 ^a^ ± 6.06	403.63 ^a^ ± 3.66	123.32 ^a^ ± 1.49	95.35 ^b^ ± 0.82	43.07 ^a^ ± 2.58	26.74 ^a^ ± 2.74	5.09 ^b^ ± 0.04	2.76 ^ab^ ± 0.18	2.06 ^b^ ± 0.07	0.56 ^a^ ± 0.01	0.16 ^d^ ± 0.02
	Genotype 9 (LYG)	481.11 ^bc^ ± 6.70	373.31 ^b^ ± 5.47	121.81 ^a^ ± 1.64	110.78 ^a^ ± 6.38	42.97 ^a^ ± 0.74	23.90 ^ab^ ± 0.66	5.78 ^a^ ± 0.07	2.96 ^a^ ± 0.01	2.63 ^a^ ± 0.04	0.54 ^a^ ± 0.00	0.23 ^d^ ± 0.02
	Genotype 16 (LYG)	456.41 ^c^ ± 20.07	337.84 ^c^ ± 13.54	107.96 ^b^ ± 4.89	81.16 ^b^ ± 3.31	41.08 ^ab^ ± 0.78	21.54 ^bc^ ± 0.98	4.48 ^c^ ± 0.19	2.36 ^b^ ± 0.14	1.63 ^d^ ± 0.06	0.42 ^c^ ± 0.02	0.26 ^cd^ ± 0.05
N effect (%)		+4.85 **	+9.45 ***	+14.59 ***	+18.55 ***	+14.86 ***	+31.44 ***	+9.39 ***	+7.98 *	(ns)	+3.69 *	+84.77 ***
*Two-way ANOVA*												
*p*-value (G effect)		**<0.001**	**<0.001**	**<0.001**	**<0.001**	**<0.001**	**<0.001**	**<0.001**	**<0.001**	**<0.001**	**<0.001**	**<0.001**
*p*-value (T effect)		**0.008**	**<0.001**	**<0.001**	**<0.001**	**<0.001**	**<0.001**	**<0.001**	**0.023**	0.25	**0.033**	**<0.001**
*p*-value (G × T)		**<0.001**	0.107	0.08	0.51	**0.046**	**0.001**	**<0.001**	**0.001**	**<0.001**	**0.008**	**<0.001**

Within each treatment, letters indicate significant differences between genotypes at *p* < 0.5. *, ** and *** are significant at *p* < 0.05, *p* < 0.01 and *p* < 0.001, respectively. ‘ns’ means insignificant at *p* < 0.05. Values in bold indicate significance (*p* < 0.05). HYG: High-yielding genotypes (grey shadowed), LYG: Low-yielding genotypes, G: Genotype, T: Treatment.

**Table 5:** Polyphenol fractions and anti-radical activity of 6 wheat genotypes fertilized by two nitrogen levels (High and Low). Means are followed by standard error (n = 4).

		Soluble Polyphenols		Hydrolysable Polyphenols		Total Polyphenols	Bio-Accessible Polyphenols		Anti-Radical Activity ^†^	
N Level	Genotypes	mg Gallic Acid/100 g Flour	% of Total Polyphenols	mg Gallic Acid/100 g Flour	% of Total Polyphenols	mg Gallic Acid/100 g Flour	mg Gallic Acid/100 g Flour	% of Total Polyphenols	Remaining µmol DPPH at Steady State	%
High N	Genotype 18 (HYG)	224 ^b^ ± 6.77	19	928 ^a^ ± 33.66	81	1152 ^a^ ± 31.00	540 ^abc^ ± 13.13	47	0.220 ^c^ ± 0.004	55
	Genotype 6 (HYG)	172 ^c^ ± 6.12	19	719 ^b^ ± 27.31	81	892 ^b^ ± 26.86	586 ^a^ ± 21.91	66	0.212 ^c^ ± 0.006	57
	Genotype 10 (HYG)	200 ^bc^ ± 8.25	21	745 ^b^ ± 30.83	79	949 ^b^ ± 41.17	552 ^ab^ ± 20.74	58	0.215 ^c^ ± 0.003	56
	Genotype 3 (LYG)	294 ^a^ ± 9.68	24	924 ^a^ ± 35.81	76	1218 ^a^ ± 36.94	575 ^ab^ ± 14.59	47	0.206 ^c^ ± 0.009	58
	Genotype 9 (LYG)	265 ^a^ ± 17.90	23	905 ^a^ ± 85.29	78	1159 ^a^ ± 80.31	531 ^bc^ ± 23.40	46	0.257 ^b^ ± 0.006	48
	Genotype 16 (LYG)	205 ^bc^ ± 17.08	18	924 ^a^ ± 52.25	82	1129 ^a^ ± 45.49	494 ^c^ ± 13.87	44	0.278 ^a^ ± 0.009	44
Low N	Genotype 18 (HYG)	297 ^ab^ ± 19.29	32	619 ^a^ ± 40.72	68	916 ^a^ ± 20.35	543 ^cd^ ± 9.33	59	0.248 ^a^ ± 0.002	50
	Genotype 6 (HYG)	297 ^a^ ± 14.89	34	573 ^a^ ± 25.42	66	873 ^ab^ ± 17.10	517 ^d^ ± 21.13	59	0.243 ^ab^ ± 0.005	51
	Genotype 10 (HYG)	293 ^a^ ± 12.58	34	559 ^a^ ± 36.03	66	851 ^ab^ ± 51.15	541 ^cd^ ± 14.89	64	0.253 ^a^ ± 0.011	49
	Genotype 3 (LYG)	284 ^ab^ ± 12.52	32	602 ^a^ ± 50.13	68	886 ^ab^ ± 45.07	601 ^ab^ ± 14.89	68	0.206 ^c^ ± 0.009	58
	Genotype 9 (LYG)	255 ^b^ ± 5.55	29	622 ^a^ ± 51.08	71	876 ^ab^ ± 52.72	647 ^a^ ± 18.43	74	0.219 ^c^ ± 0.006	56
	Genotype 16 (LYG)	263 ^ab^ ± 5.48	33	525 ^a^ ± 23.87	67	788 ^b^ ± 23.38	587 ^bc^ ± 21.87	74	0.223 ^bc^ ± 0.010	55
N effect (%)		−19.36 ***		+47.03 ***		+25.21 ***	−4.62 *		ns	
*Two-way ANOVA*										
*p*-value (G effect)		**<0.001**		**0.01**		**<0.001**	**0.02**		**<0.001**	
*p*-value (T effect)		**<0.001**		**<0.001**		**<0.001**	**0.01**		0.91	
*p*-value (G × T)		**<0.001**		0.06		**0.002**	**<0.001**		**<0.001**	

Within each treatment, letters indicate significant differences between genotypes at *p* < 0.5. * and *** are significant at *p* < 0.05 and *p* < 0.001, respectively. ‘ns’ means insignificant difference at *p* < 0.05. Values in bold indicate significance (*p* < 0.05). ^†^ corresponding to 2.4 mg flour or 2.4–2.6 mg bread at flour basis that consumed DPPH when 0.494 µmol of the free radical are initially available to react. The plateau was decided at 120 min of reaction. HYG: High-yielding genotypes (grey shadowed), LYG: Low-yielding genotypes.

## Supplementary Materials

The following is available online at https://www.mdpi.com/2223-7747/9/12/1636/s1, Table S1: Pedigree and selection history of the 20 durum wheat genotypes obtained from the International Maize and Wheat Improvement Center (CIMMYT, Mexico).
