# Supplementary material for: Durum Wheat Grain Yield and Quality under Low and High Nitrogen Conditions: Insights into Natural Variation in Low- and High-Yielding Genotypes
Source: Plants (Basel). 2020 Nov 24;9(12):1636. doi: 10.3390/plants9121636 (PMC7760076; doi:10.3390/plants9121636)
Supplement: Supplementary file 1 [file plants-09-01636-s001.pdf]

Table S1. Pedigree and selection history of the 20 durum wheat genotypes obtained from the International Maize and Wheat Improvement Center (CIMMYT, Mexico).

Genotypes	Pedigree
1	ALTAR 84/STINT//SILVER_45/3/GUANAY/4/GREEN_14//YAV_10/AUK/5/GUAYACAN INIA/… (CDSS11B00002S-0133Y-055M-2Y-0M)
2	BYBLOS/7/WID22256/5/ALTAR 84/STINT//SILVER_45/3/GUANAY/4/GREEN_14//YAV_10/… (CDSS12Y00252S-09Y-018M-2Y-0M)
3	BYBLOS/7/WID22256/5/ALTAR 84/STINT//SILVER_45/3/GUANAY/4/GREEN_14//YAV_10/… (CDSS12Y00252S-09Y-018M-9Y-0M)
4	CBC 509 CHILE/6/ECO/CMH76A.722//BIT/3/ALTAR 84/4/AJAIA_2/5/KJOVE_1/7/AJAIA_12/… (CDSS12Y00717T-035Y-017M-1Y-0M)
5	SILVER_14/MOEWE//BISU_1/PATKA_3/3/PORRON_4/YUAN_1/9/USDA595/3/D67.3/RABI//CRA/4/… (CDSS11B00068S-066Y-064M-10Y-0M)
6	CBC 509 CHILE/6/ECO/CMH76A.722//BIT/3/ALTAR 84/4/AJAIA_2/5/KJOVE_1/7/AJAIA_12/… (CDSS11B00319T-044Y-028M-39Y-0M)
7	ALTAR 84/STINT//SILVER_45/3/GUANAY/4/GREEN_14//YAV_10/AUK/5/SOMAT_4/INTER_8/6/… (CDSS11B00351T-072Y-051M-20Y-0M)
8	ADAMAR_15//ALBIA_1/ALTAR 84/3/SNITAN/4/SOMAT_4/INTER_8/5/SOOTY_9/RASCON_37/6/… (CDSS11B00356T-045Y-025M-41Y-0M)
9	SOOTY_9/RASCON_37//GUAYACAN INIA/11/BOOMER_33/ZAR/3/BRAK_2/AJAIA_2//… (CDSS12Y00165S-072Y-032M-23Y-0M)
10	SILVER_14/MOEWE//BISU_1/PATKA_3/3/PORRON_4/YUAN_1/9/USDA595/3/D67.3/RABI//CRA/4/… (CDSS11B00138S-078Y-055M-15Y-0M)
11	GERUFTEL-1//GUAYACAN INIA/2*SNITAN (CDSS10Y00291S-099Y-044M-5Y-2M-06Y-0B)
12	CBC 509 CHILE/SOMAT_3.1//BOOMER_18/LOTUS_4/6/SOMAT_3/PHAX_1//TILO_1/… (CDSS10Y00493T-099Y-035M-9Y-4M-06Y-0B)
13	CMH83.2578/4/D88059//WARD/YAV79/3/ACO89/5/2*SOOTY_9/RASCON_37/6/1A.1D 5+1-06/… (CDSS10B00122T-099Y-011M-4Y-0M-06Y-0B)
14	MOHAWK/6/LOTUS_5/F3LOCAL(SEL.ETHIO.135.85)/5/CHEN… (CDSS11Y00431S-099Y-032M-13Y-0M-06Y-0B)
15	MOHAWK/6/LOTUS_5/F3LOCAL(SEL.ETHIO.135.85)/5/CHEN… (CDSS11Y00438S-099Y-029M-16Y-0M-06Y-0B)
16	LABUD/NIGRIS_3//GAN/3/AJAIA_13/YAZI/10/PLATA_10/6/MQUE/4/USDA573//QFN/AA_7/3/… (CDSS07Y00042S-099Y-099M-15Y-1M-04Y-0B)
17	MÂALI/5/LOTUS_5/SORD_1/3/CANELO_8//SORA/2*PLATA_12/4/YAZI_1/AKAKI_4//SOMAT_3/3/… (CDSS06B00054S-099Y-099M-1Y-1B-04Y-0B)
18	MÂALI/6/MUSK_1//ACO89/FNFOOT_2/4/MUSK_4/3/PLATA_3//CREX/ALLA/5/OLUS*2/ILBOR//… (CDSS07Y00784D-2B-07Y-07M-7Y-4B-04Y-0B)
19	YAZI_1/AKAKI_4//SOMAT_3/3/AUK/GUIL/GREEN/5/2*NETTA_4/DUKEM_12//RASCON_19/3/SORA/… (CDSS04B00346T-0TOPY-3Y-0M-4Y-3Y-0B)
20	C F4 20 S/4/YAZI_1/AKAKI_4//SOMAT_3/3/AUK/GUIL//GREEN/5/CANELO_9.1//SHAKE_3/… (CDSS09B00035S-099Y-069M-8Y-4M-06Y-0B)
